# Effect of Primary Care Center Characteristics, Healthcare Worker Vaccination Status and Patient Economic Setting on Patient Influenza Vaccination Coverage Rates

**DOI:** 10.3390/vaccines11061025

**Published:** 2023-05-25

**Authors:** Christian Bengoa Terrero, Marian Bas Villalobos, Ana Pastor Rodríguez-Moñino, María Dolores Lasheras Carbajo, Julián Pérez-Villacastín, María Jesús García Torrent, Rafael Sánchez-del-Hoyo, Eneko Bengoa San Sebastian, Alberto García Lledó

**Affiliations:** 1 Servicio de Cardiología, Hospital Clínico San Carlos, 28040 Madrid, Spain; 2 Gerencia Asistencial de Atención Primaria, Servicio Madrileño de Salud, 28035 Madrid, Spain; 3 Dirección General de Salud Pública, Consejería de Sanidad de la Comunidad de Madrid, 28009 Madrid, Spain; 4 Departamento de Medicina, Facultad de Medicina, Universidad Complutense de Madrid, 28040 Madrid, Spain; mariaj58@ucm.es; 5 Unidad de Apoyo Metodológico a la Investigación, Hospital Clínico San Carlos, IdISSC, 28040 Madrid, Spain; 6 Fundación Interhospitalaria para la Investigación Cardiovascular (Fundación FIC), 28008 Madrid, Spain; 7 Servicio de Cardiología, Hospital Príncipe de Asturias, Alcalá de Henares, 28805 Madrid, Spain

**Keywords:** influenza, vaccination, healthcare workers, deprivation, uptake, socioeconomic status, correlation, workload

## Abstract

Background: Reaching the public health organizations targets of influenza vaccination in at-risk patient groups remains a challenge worldwide. Recognizing the relationship between the healthcare system characteristics and the economic environment of the population with vaccination uptake can be of great importance to improve. Methods: Several characteristics were correlated in this retrospective ecological study with data from 6.8 million citizens, 15,812 healthcare workers across 258 primary care health centers, and average income by area of the care center in Spain. Results: No correlation between HCW vaccination status and patient vaccination was found. A weak negative significant correlation between the size of the population the care center covers and their vaccination status did exist (6 mo.–59 yr., *r* = 0.19, *p* = 0.002; 60–64 yr., *r* = 0.23, *p* < 0.001; ≥65 yr., *r* = 0.23, *p* ≥ 0.001). The primary care centers with fewer HCWs had better uptake in the at-risk groups in the age groups of 60–64 yr. (*r* = 0.20, *p* = 0.002) and ≥65 (*r* = 0.023, *p* ≥ 0.001). A negative correlation was found regarding workload in the 6 mo.–59 yr. age group (*r* = 0.18, *p* = 0.004), which showed the at-risk groups that lived in the most economically deprived areas were more likely to be vaccinated. Conclusions: This study reveals that the confounding variables that determine influenza vaccination in a population and in HCWs are complex. Future influenza campaigns should address these especially considering the possibility of combining influenza and SARS-CoV-2 vaccines each year.

## 1. Introduction

Influenza has a considerable impact on at-risk groups across the globe. It is a highly contagious respiratory illness and has proved to be a significant public health issue, affecting millions of people globally each year. The disease can range in severity from mild to severe and, in some cases, can lead to hospitalization and death, particularly in high-risk populations such as young children, the elderly, and people with underlying medical conditions. Influenza can have a significant impact on an individual’s quality of life and can result in lost workdays and decreased productivity [1,2,3,4,5].

Influenza also puts strain on healthcare systems each season as patients seek medical attention, including hospitalization or intensive care. During the 2011–2012 Spanish winter season, the excess mortality attributable to influenza was between 8110 and 10,872 [6] and, during the 2017–2018 winter, even higher, with close to 15,000 estimated deaths [7].

The economic burden is also noteworthy; the European Union estimates the direct and indirect costs of influenza is EUR 11.8 billion. Just in Spain, depending on the variables that the studies include (sickness absence, hospitalizations, consultations, or emergency visits), the economic impact ranges between EUR 250 million and one billion per year [8,9,10,11]. This is a considerable burden especially when effective preventative pharmacological interventions exist, as is the case with the influenza vaccine.

Public health organizations, such as the Center for Disease Control, the European Center for Disease Control, and the World Health Organization, all recommend that influenza vaccination reach 75% in at-risk groups [12]. Healthcare workers (HCWs), pregnant women, patients with pre-existing conditions, and people over 65 years old all classify as such. Even with the ever-increasing evidence in favor of influenza vaccination, attaining the targets in at-risk groups is rarely achieved in European countries [12,13].

The SARS-CoV-2 pandemic has heightened awareness for vaccination and has pushed up influenza vaccination across the globe [14,15,16,17,18,19,20]. However, even with this new drive, which is likely to eventually subdue, health systems struggle to reach their targets. During the first COVID-19 pandemic influenza season (2020–2021), only three of the 38 OECD countries, Denmark, Korea, and Chile, surpassed 75% uptake in people over 65 years of age. Spain was twelfth on the list with 67.7% uptake in people over 65 years of age that same year [14].

Several confounding variables come into play when individuals seek vaccination. Patients report that endorsement from their medical professional encourages them to seek influenza vaccination [21,22]. Since HCWs are those responsible for the health of individual people and they are recognized as being the most scientifically competent, their voice on the issue changed individual behaviors one-way or the other. This is particularly important considering HCWs who are vaccinated are more likely to recommend vaccination to their patients [23,24,25,26,27].

Economic factors also play a role in determining individual health and access to healthcare. The most economically deprived areas have worse health and paradoxically have worse access to care [28,29,30,31,32,33,34,35,36]. This is known within the healthcare field as the Reverse Care Law. Research has been carried out specific to influenza vaccination and socioeconomic status, but the results have been contradictory with some studies resulting in both more affluent individuals having lower uptake and others concluding just the opposite [37,38]. In a 2006 study that surveyed 92,101 households across 11 European countries, the data reveal that gender, household income, household size, education level, and population size of the living residence significantly influences the likelihood of becoming vaccinated; however, the results varied and were dependent on each country. More recently, a systematic review on the impact of economic deprivation and vaccination status argued the relationship is context/country specific [39].

It is possible that other factors play a role in vaccination rates in at-risk groups such as the workload of HCWs. As health systems try to achieve maximum return on their employees by only allowing for short consultations, this likely impacts HCWs’ ability to address preventative health interventions. We found no published evidence that studies the relationship of HCW workload and the vaccination status of patients.

The purpose of our study was to analyze the effect of the characteristics of the primary care health centers and the economic environment on the patient vaccine uptake, considering the size of the care center, the vaccination acceptance of HCW, and their workload.

## 2. Materials and Methods

### 2.1. Setting, Study Design, and Population

This is a retrospective ecological study that includes the whole population covered by the public health care system of the Autonomous Community of Madrid (ACM), Spain, during the influenza season of 2020–2021. Since 100% of citizens are covered by this system [40], a total 6,751,251 people form the initial group. The population has access to primary care at no cost through the 15,812 HCWs distributed across the 262 care centers. As is often the case, one municipality can be covered by several care centers.

### 2.2. Data Sources and Definitions

Vaccination status by population age group and care center for the 2020–2021 influenza season was provided by the Primary Care Directorate of the ACM. The vaccination rate of the population was divided into three asymmetric groups: from 6 months to 60 years of age, between 60 and 64 years, and those aged 65 or older. This age grouping is how the ACM provides the universally targeted group, people over 65, and seek vaccination in those between 60 and 65 years of age.

The data regarding the total population covered per care center and the number of HCWs employed at each care center were also provided by the Primary Care Directorate.

The workload per HCW was estimated as the average assigned population per HCW. This is a division between the population covered in a care center divided by the number of employed HCWs.

The data for the HCWs’ vaccination statuses were provided by the Public Health Directorate of the ACM for that same year. The definition of HCWs for this study is broad as it includes not just clinicians but also all support staff that are employed at the care centers.

The ACM has 180 municipalities (cities or towns) with the largest population residing within the City of Madrid (3.3 million. 2021), which, in turn, is divided into 21 districts. Due to its comparative size, it will be the only municipality divided into districts in this study.

The economic information of each care center social environment, considering its municipality or district, was downloaded from the Spanish National Institute of Statistics for the most recent available year (2019) [41]. The mean income per person and median income per consumption unit were retrieved. This is the gold standard for average household economic status, as it combines survey data and governmental administrative information [41]. A consumption unit is used to correct for differences in consumption needs between households of different sizes and composition to arrive at comparable results [42]. As needs increase with each additional household member, although not in a proportional way due to economies of scale, equivalence scales are used to reflect the needs of different compositions of households, assigning a value to each household member in proportion to its needs. A value of 1 is assigned to the first adult, 0.5 to additional adults, and 0.3 to children under 14. This is already calculated by the Spanish National Institute of Statistics.

### 2.3. Data Manipulation and Statistical Analysis

Each care center was allocated to either one municipality or one of the City of Madrid’s 21 districts, thus having a matching mean income per person and a median per consumption unit data point.

Thus, for each care center, the uptake by age group was correlated with the number of people served, the total number of HCWs, influenza uptake in HCWs, estimated workload per HCW, mean income per person, and median income per unit of consumption.

The data were analyzed using IBM SPSS Statistics v.26. A level of 0.05 was chosen for the statistical significance. The Pearson correlation coefficient was used for all correlations with the exception of the mean income, which is an asymmetric variable. In this case, the Spearman correlation was used.

Ethical approval for this study was granted by the Ethical Committee of the Hospital Clínico San Carlos under code C.I. 21/118-E.

## 3. Results

### 3.1. Influenza Vaccine Uptake in the Population Groups

A total of 262 primary care centers were identified. Due to the absence of data or incongruences between the two different public databases used, four care centers were excluded, leaving a total of 258 care centers. The 258 care centers served a population of 6,839,539, of whom 1,170,076 were 65 years or older and 382,045 were from 60 to 64 years old.

Among the target population group of 65 years or older, the average uptake was of 60.65%. In this age group, the highest uptake in a single primary care center was 99.58%, and the lowest 42.34%. Table 1 shows the vaccine uptake for different age and sex groups. The female sex is associated with a significantly greater influenza uptake in all age groups

The vaccine uptake in people 65 years of age or older showed a positive correlation with the vaccination uptake of the other age groups, with *r* = 0.82 (*p* < 0.001) for the interval between 60 and 64 and *r* = 0.57 (*p* < 0.001) for those between 6 months and 59 years.

### 3.2. Relationship between Healthcare Workers and Corresponding Population Uptakes

In total, 15,812 HCWs were employed at 258 care centers. The average number of HCWs per care center was 60 (median = 58). The largest care center employs 157 workers and the least 13. Influenza vaccination uptake during the 2020–2021 season among HCWs was of 56.61%, with the highest uptake in a single center of 80% and the lowest of 20%. No correlation was found between the number of staff employed at a care center and the vaccination status of its HCWs (*r* = −0.05, *p* = 0.43). A weak negative correlation was found between the workload of the HCWs and their own vaccination statuses, but it was not significant (*r* = −0.10, *p* = 0.10). No correlation was found between the HCWs’ vaccination status and the vaccination statuses of their attended population.

### 3.3. Primary Care Centre Characteristics and Vaccination Uptake of the Corresponding Population

The average total population per care center was 26,510 (range: 2457–61,921) (excludes age < 6 months). Care centers that cover a larger population have lower uptakes in the population as a whole (*r* = −0.34, *p* < 0.001). This relationship exists in all age groups when correlating like with like as shown in Table 2, i.e., care centers with a larger ≥ 65 group have a lower uptake in that group.

The average assigned population (or workload) per HCW was 447 (range 111–1334). In the primary care centers where HCWs had a smaller workload, a negative correlation was found only with the 6 mo.–59 yr. age group. (6 mo.–59 yr., *r* = −0.18 *p* = 0.004; 60–64 yr., *r* = −0.12, *p* = 0.06; ≥65 yr., *r* = −0.08, *p* = 0.19).

A negative correlation was found between the number of HCWs employed at a care center and the vaccination status of the individuals ≥ 65 (*r* = −0.23, *p* < 0.001) or between 60 and 64 (*r* = 0.20, *p* = 0.001) years, but not individuals aged 6 mo.–59 yr. (*r* = 0.09, *p* = 0.16). This could mean that care centers with fewer HCWs are slightly better at achieving higher coverage rates.

### 3.4. Economic Environment and Vaccination Uptake of Attended Population

The lowest mean income per person at a care center for 2019 was EUR 9494 a year and the highest EUR 27,634 (mean: EUR 15,465). For the median income per consumption unit, the lowest was EUR 12,950 and the highest EUR 31,850 (mean: EUR 20,018).

A negative correlation was found between the influenza uptake in all the age groups and income. The strongest negative correlation can be found between the mean income per person and influenza vaccine uptake in the ≥65 group as shown in Table 3.

## 4. Discussion

This study encompasses a set of primary care center characteristics and economic variables that could influence the acceptance of vaccination in patients in the ACM, which is a Spanish region with universal health coverage. This allowed us to analyze a large sample size with a relatively homogeneous health care system.

While the mean uptake of the vaccine in all care centers (56.61%) was reasonable in the context of other European countries [14], the wide range observed between care centers, with some reaching 20% uptake and others 80%, suggests enormous room for improvement is possible and provides meaning to studies such as this one aimed at investigating the causes of these variations. A certain level of variation is to be expected, but this puts a special focus on care centers that are seemingly underperforming. A case could be made that it is these lower performing primary care centers that drive down the overall average and could therefore be the target of specific strategies to increase uptake in the population.

As expected, there is a correlation in vaccination uptake between the age groups. If the population of ≥65 becomes vaccinated, it seems coherent that the other age groups from the same care center would also become vaccinated. This confirms that the there are certain area specific variables that play a role when driving people to become vaccinated, but it does not reveal the causes as to why certain areas are more vaccinated than others. Several significant negative correlations were found that help understand these causes.

In essence, smaller care centers that attended smaller populations in less wealthy areas obtained better vaccination rates. However, no relationship was found between the influenza vaccine uptake in HCWs and the vaccination status of the attended population. A significant correlation was found between the calculated HCW workload and the vaccination status of the attended population in the 6 months–59-year-old group. It is important to note that the data provided are for an influenza season during the SARS-CoV-2 pandemic, when health systems were under strain and before a coronavirus vaccine was available.

The absence of correlation between the vaccination status of HCWs and the vaccination status of the age groups, particularly in the ≥65, was unexpected. Prior evidence suggests vaccinated professionals are more likely to recommend influenza vaccination to their patients [23,24,25,26,27]. Even with this new evidence, this is likely still true. Rather, we put into question whether HCWs in the ACM recommended influenza vaccination when they encountered patients that season. This could be explained by the lack of time and personal contact primary care HCWs had during the SARS-CoV-2 pandemic during consultations, meaning they purely offered reactive medicine rather than proactive preventative medicine.

Another motive for this result may be that messaging for influenza vaccination is coordinated regionally; therefore, it is messaged homogeneously to all at-risk groups allowing little room for professionals to play a role, or it makes it easier for them to relax in providing advice. Additionally, people were exceptionally aware from the COVID-19 pandemic and had formed opinions on vaccination, meaning fewer people had an ambivalent position, which is the group where the HCW recommendations can have the biggest impact.

We expected that a better ratio of HCWs per population (HCW workload) could facilitate vaccination recommendation, but, surprisingly, this was not found in the 60–64 nor the ≥65 age group. Nonetheless, our results suggest that smaller health centers have a stronger rapport with their patients since a significant negative correlation was found with regard to care center population size. A cause for this could be the closer contact HCWs have with patients in smaller towns and rural areas. We must also consider that our study was performed during the first year of the SARS-CoV2 pandemic, and many circumstances could have affected the results. For example, the confinement was stricter in urban areas with generally higher incomes.

Our study reveals that those in the most economically deprived areas are more likely to become vaccinated against influenza. There may be several causes for this.

People from the most economically deprived areas of the ACM are more likely to have frontline essential jobs, use public transport, travel further, share their home with more people, and have therefore been disproportionally affected by COVID-19, both in infections and mortality [43,44]. This could have created a stronger awareness in favor of vaccination.

Previous research on the role of socioeconomic status in predicting influenza vaccination paints a varied picture. Similar to our article, a comprehensive Italian study on health literature and socioeconomic status from 2022, with a smaller sample than the one analyzed in our article (*n* = 3278), found that those who were not employed or had a poor financial status were significantly more likely to be vaccinated against influenza [38]. A Columbian study, with 2000 people older than 60, reached the same conclusion [45].

In another Italian study (*n* = 25,183) with over 65 year olds, the authors state their surprise after they found that influenza vaccination was more prevalent in lower social classes (65.1%, AOR 1.2, 95% CI 1.1, 1.3, *p*  <  0.01) than in upper social classes (56.9%) [37]. This same study [37] also sheds light on why this may be the case: lower income individuals have worse health and worse health self-perception, and consequentially seek and receive recommended vaccinations more insistently. Other research also finds a significant link between worse health and higher influenza uptake [46,47]. Worse health could cause more visits to the care center, a fact that facilitates contact with HCWs and recommendations during influenza vaccine campaigns. The relationship between individual health and socioeconomic status is well evidenced in the medical literature [28,29,30,31,32,33,34,35,36]. This could help explain the results in our study.

On the contrary, several studies conclude that a higher socioeconomic status links favorably with vaccination status. This conflicting evidence is well described in a systematic review by Vukovic et al. (2020) [39]. Most of the compiled articles show that the most economically deprived tend to have lower influenza vaccination coverage; however, nearly all the evidence came from the United Kingdom, and the articles stating the opposite were well supported. In a cross-sectional study (2020), with a sample of 19,793 US adults with atherosclerotic cardiovascular disease, low-income households had significantly lower rates of influenza vaccination [48]. This raises a question on the role universal/public health systems play in encouraging people to become vaccinated as well as the accessibility of healthcare overall. The data are not contradictory but specific to each region.

We consider the strongest cause for the negative correlation between vaccination status in the 60–64 and ≥65 groups with income is due to those being in the most economically deprived areas being in worse health and therefore being more likely to perceive a need to become vaccinated against influenza. However, this is an interpretation of the data since no data on health status were included.

Our study had limitations. First, we had no information on the demographic characteristics of age or sex for HCWs that could affect their vaccination rate. Secondly, our study did not include health data of the age groups, such as the prevalence of chronic diseases from each care center, to neutralize this confounding variable. If this information was included, we would be able to discern if influenza vaccination by care center was due to economic or health factors, or, most likely, a combination of the two. Similarly, data on at-risk groups or individuals with an inclination for influenza vaccination would be helpful, especially for a comprehensive analysis of all age groups or even by disease type, e.g., looking at whether patients with diabetes in the most economically deprived areas have higher uptake than those in the richer areas.

Other confounding variables have also been omitted, including the population education level, household size, and proportion of the elderly, which would arguably also play a role in vaccination uptake.

Finally, as previously stated, it is important to note that the data available here are for the 2020–2021 influenza season, which was during the SARS-CoV-2 pandemic. This has likely affected the results in different ways. While the lack of personal contact of HCWs with patients due to confinement and the saturation of the health care system could have decreased the effect of HCW recommendation, the pandemic could have increased everyone’s perception of the preventative value of vaccines.

## 5. Conclusions

Our results show that the ratio of HCWs per population served (workload) or the size of the primary care center do not benefit the vaccination uptake in the target vaccination groups (>60 years old). In fact, better uptakes have been observed in smaller care centers. Additionally, unexpectedly, vaccination uptake is higher in poorer areas most likely due to the most economically deprived having worse health therefore seeking and being recommended influenza vaccination more frequently. We have observed a wide range of influenza vaccination uptake, from 20% to 80%, among the primary care centers within the same health care system, which is a difference that is hard to explain even considering the influence of social or economic variables and the difficulties during the SARS-CoV2 pandemic. Our results suggest that closer contact with the health care system in smaller towns and for less wealthy subjects with worse health statuses facilitates vaccination, and also support the need of HCW training in vaccination recommendation, beyond general public information campaigns.

## Figures and Tables

**Table 1:** Influenza vaccine uptake by age group and sex.

	Male		Female		Total		
Age Group	Sample	% Vaccinated	Sample	% Vaccinated	Sample	% Vaccinated	*p*-Value
≥65	484,970	61.2	685,106	60.3	1,170,076	60.7	<0.001
60–64	177,980	34.6	204,065	39.6	382,045	37.3	<0.001
6 mo.–59 yrs.	2,614,985	7.6	2,672,433	10.3	5,287,418	9.0	<0.001

**Table 2:** Correlation between primary care center characteristics and the uptake of the age groups.

	6 Months–59 Years Old	60–64 Years Old	≥65 Years Old
Population covered by the primary care center	−0.19 (*p* = 0.002)	−0.23 (*p* < 0.001)	−0.23 (*p* < 0.001)
Number of HCWs employed at the primary care center	−0.09 (*p* = 0.16)	−0.20 (*p* = 0.002)	−0.23 (*p* < 0.001)
HCW workload	−0.18 (*p* = 0.004)	−0.12 (*p* = 0.06)	−0.08 (*p* = 0.19)

**Table 3:** Correlation coefficients between groups in influenza vaccination uptake and income type.

Group	Mean Income per Person	Median Income per Consumption Unit
6 months–59 years old	−0.23 (*p* < 0.001)	−0.18 (*p* = 0.003)
60–64 years old	−0.38 (*p* < 0.001)	−0.26 (*p* < 0.001)
≥65 years old	−0.41(*p* < 0.001)	−0.32 (*p* < 0.001)

## Data Availability

The data presented in this study are available on request from the corresponding author.
